# Container-based sanitation in Nairobi’s Mukuru: roles, power, challenges and strategies

**DOI:** 10.3389/fpubh.2025.1674329

**Published:** 2025-12-04

**Authors:** Ivy Chumo, Caroline Karani, Joy Riungu, Lilian Mukiri Kirimi

**Affiliations:** 1 African Population and Health Research Center, Nairobi, Kenya; 2 Meru University of Science and Technology, Meru, Kenya

**Keywords:** container based sanitation, power dynamics, urban sanitation, governance, equity, community participation, public health policy

## Abstract

Rapid urbanization in the developing world has exacerbated the crisis of poor sanitation within sprawling informal settlements. Container-Based Sanitation (CBS) has emerged as a promising, alternative solution designed to fill these critical infrastructural deficits where conventional sewage systems are absent. Despite its apparent potential, there is a recognized gap in the contextual understanding of CBS implementation, particularly concerning the influence of local social and political dynamics, which ultimately hinders effective scale and sustainable programming. This qualitative study aimed to explore the intricate factors surrounding the implementation of CBS in an urban slum, specifically investigating the technology’s operational roles, the influence of existing power structures, the key challenges encountered, and viable strategies for enhancing its adoption within an informal settlement in Nairobi, Kenya. Data collection utilized in-depth interviews (IDIs) with 25 key participants, including residents, local community leaders, and relevant government officials working in the Mukuru Kwa Reuben informal settlement. A purposive sampling method guided participant selection, and the collected qualitative data was rigorously analyzed using a framework analysis approach. The study’s findings indicate that CBS was perceived positively as a cost-effective and safe solution for improving household sanitation. However, its widespread scalability was significantly limited by a series of entrenched contextual challenges. The challenges stemmed from a inadequate governance, power structures and coordination of governance and coordination, including inaccurate data and failed consultation, which limited project success. Financially, the sector was underfunded and deprioritized, leading to an inequitable, high-cost user-fee model that burdened residents. A consistent lack of community involvement undermined the long-term sustainability and adoption of the interventions. The research concludes that scaling CBS successfully requires transcending purely technical considerations. The findings will contribute to policy-making in Nairobi’s Special Planning Area framework and broader debates on urban sanitation financing. Future implementation strategies should focus on establishing collaborative governance models and integrating local leadership to navigate and mitigate existing power dynamics, thereby addressing the systemic, contextual barriers required for achieving sustainable and equitable sanitation coverage.

## 1 Introduction

Rapid urbanization in the developing countries leaves a little space for proper infrastructure, including sanitation access and service delivery (1, 2). Unplanned urban settings pose particular challenges such as difficult access; lack of space for construction of sanitation facilities, lack of land ownership and title deeds, often challenging physical and topographical conditions; lack of water supply; and/or regular exposure to flooding (3, 4). The construction of toilets, faecal emptying, and transportation services is challenging due to insufficient space for facilities and the lack of legally designated land for excreta disposal or access for emptying trucks (3). However, even when toilets apertures allow for emptying, toilets collapse due to lack of reinforced structure. The cost of faecal emptying in such areas could be high due to strains in access to the latrines. In such circumstances, the traditional approach to urban sanitation, premised on extending sewerage networks and building wastewater treatment (WWT) plants, may not be sufficient to deliver citywide sanitation services for all (4). Approaches are needed to meet contextual sanitation challenges so that the population living in informal settings, who are often among the most vulnerable, can benefit from adequate sanitation services (5, 6). As such, systems that are capable of addressing and withstanding the challenges are needed to ensure universal access to sanitation even in informal settlements.

Urban political ecology research highlights profound governance challenges in sanitation management across African cities. Political relationships shape sanitation management, leading to the rhetorical adoption of good governance policies over meaningful implementation in Accra, Addis Ababa, Maputo, and Ouagadougou urban areas (31). Institutionalized democracy can create participatory spaces with narrow mandates and limited capacity (32), and political constraints often rooted in local government structures disproportionately affect low-income populations (40). Jenkins (32) examines water and sanitation governance in peri-urban Dodowa, Ghana, finding that institutionalized local democracy created participatory spaces with narrow mandates and limited capacity, while community water and sanitation committees failed to foster inclusive participation. Odili and Sutherland (39) analyze Durban’s evolving sanitation landscape through a governmentality framework, identifying a shift toward more diverse, context-specific approaches that deepen state engagement with communities and technology. Nunan and Satterthwaite (40) highlight inadequate water, sanitation, and waste collection across nine cities, emphasizing political constraints rooted in local government structures and relationships with citizen groups that particularly affect low-income populations.

In Kenya, sewer system is prioritized as the formal system of sanitation provision. Yet, the Kenya Water Services Regulatory Board (WASREB) (42) report indicates that about 15% of urban population is connected to a sewerage system while the rest of the urban population uses onsite technologies. However, high population density, unplanned nature of settlements in slums, low economic status and poor access to water and sanitation services could make sewer connection in slums nearly impossible. Despite their widespread use, onsite technologies are not linked to a sanitation chain for safe disposal or reuse (WASREB, 42). Unsafe disposal of faecal matter from onsite sanitation systems could expose the slum population to diarrheal diseases, which are among the top causes of diarrheal-related morbidities in Kenya (33). Besides disposal concerns, onsite sanitation facilities are mostly shared by several households and have been reported to be of poor quality in terms of cleanliness, privacy and safety (34) due to low cooperation from households, adherence of cleaning schedules and poor toilet designs.

In 2018, the Joint Monitoring Progress report formally recognized Container-based sanitation (CBS) system as a type of improved sanitation facility (7). Container-based sanitation approaches have therefore emerged as an alternative service approach for the urban poor; to those served by sewers or by on-site sanitation (OSS) systems (4). The CBS system consists of an end-to-end service; one provided along the whole sanitation service chain, that collects excreta hygienically from toilets designed with sealable, removable containers and strives to ensure that the excreta is safely treated, disposed of, and reused (4, 8). Rather than having to build a sanitation facility, households (or public toilet operators) can sign up for a service (4, 9). The CBS service provider installs a toilet with sealable excreta containers (also referred to as cartridges) and commits to emptying them (that is, removing and replacing them with clean ones) on a regular basis (4). The importance of such services goes beyond convenience for the low-income earners to addressing challenges related to need for more space for rebuilding new latrines.

The CBS full value chain approach follows the SDG definition for “safely managed” household sanitation (7, 10). It enables addition of a certain level of dignity, comfort and ease of access for the older adults and persons with disability who would have difficulty squatting in a conventional pit latrine (11). Costs related to CBS operation, maintenance and installation reduces consumer burden (12) as compared to the ordinary sanitation options, although it may be difficult to do a direct comparison given the differences in services and cost structures. Different CBS providers charge different user fees and apply different service delivery approaches for instance on frequency and process of emptying (4). The CBS models’ monthly charges act as a convenient way for cost paying among poor households to obtain a reliable sanitation service over time as compared to the need to mobilize funds for initial connection charges, as required in the traditional sanitation options (4). In informal settlements where water availability and infrastructure are inadequate, CBS fills the gap by offering water-saving solution and cutting water cost by 6 cubic meters (m^3^)/person to 25 m^3^/person annually compared to water-flush systems that require more water, higher costs and sometimes connection to a sewer system (13, 14). Systems focusing on the poor and involving finances require a well-defined service delivery structure and an enabling environment. Without defining the role of actors, the sanitation systems might exhibit a slow growth and their operation and maintenance could be below standard due to possible mismanagement, unclear mandates, roles and regulations (4, 12).

Globally, CBS services are largely delivered by non-governmental actors in small scale services, and are either formal or informal (12, 15). Formal actors are groups or individuals with defined and structured guidelines, whereas informal actors operate without defined guidelines (16). Informal actors are diverse and may include community members or customary local governance institutions, yet they are understudied, particularly with regard to how they complement formal actors in Nairobi’s informal settlements (17, 18). Where efforts are being made to deliver basic services to an informal settlement, it is crucial that the relevant actors understand the specific context, including the role of formal and informal actors, and the efforts that are being made by informal service providers and the residents to improve the situation.

In illegal settings, formal and informal actors and the influence they have in decision making and operation of services cannot be underrated. Different actors could contribute to exclusion and exploitation of diverse groups in conditions of informality due to their power to exercise authority over services (35). A study by Anciano and Piper (36) showed that in circumstances where the government fails to provide water and sanitation services for citizens, exploitive cartels may arise to bridge the gap in service provision. In Nairobi, failure of the Government to provide water services to informal settlements facilitated the rise of cartels to supply the services to the unserved areas (37). Presence of cartels in the sanitation service provision has been associated with hiked service provision prices, insecurities, poor services, blackmails and vandalism of structures (41).

To ensure sustainable adoption of CBS, a comprehensive understanding of underlying roles of actors, power structures and challenges is needed to facilitate development of effective strategies for CBS which is the focus of this study. Besides, while container-based sanitation offers a promising solution, its implementation and scalability could be hindered by several challenges (11) related to government support, regulation and financing which require mitigation strategies for successful implementation of CBS to be realized.

Container-Based Sanitation has emerged as a promising, alternative solution designed to fill these critical infrastructural deficits where conventional sewage systems are absent. Despite its apparent potential, there is a recognized gap in the contextual understanding of CBS implementation, particularly concerning the influence of local social and political dynamics, which ultimately hinders effective scale and sustainable programming. This qualitative study aimed to explore the intricate factors surrounding the implementation of CBS in an urban slum, specifically investigating the technology’s operational roles, the influence of existing power structures, the key challenges encountered, and viable strategies for enhancing its adoption within an informal settlement in Nairobi, Kenya.

## 2 Methodology

### 2.1 Study design

The qualitative study applied in-depth interviews (IDIs) and researcher observations.

### 2.2 Study setting

The study was conducted in Mukuru Kwa Reuben informal settlements in Nairobi, Kenya. Mukuru Kwa Reuben is an extensive settlement located East of Nairobi’s city centre. It borders the industrial area and many residents work as casual laborers in the industries. It is one of the 21 villages of Mukuru, that has an estimated population of about 650,000 people (KNBS, 2019). It is prone to several challenges and risks which include sanitation, hygiene, fire, floods, insecurity, and diseases. Extreme poverty and human rights violation are prevalent. Losses due to shocks are frequent and can be massive and catastrophic (KNBS (19)).

**Figure 1:**
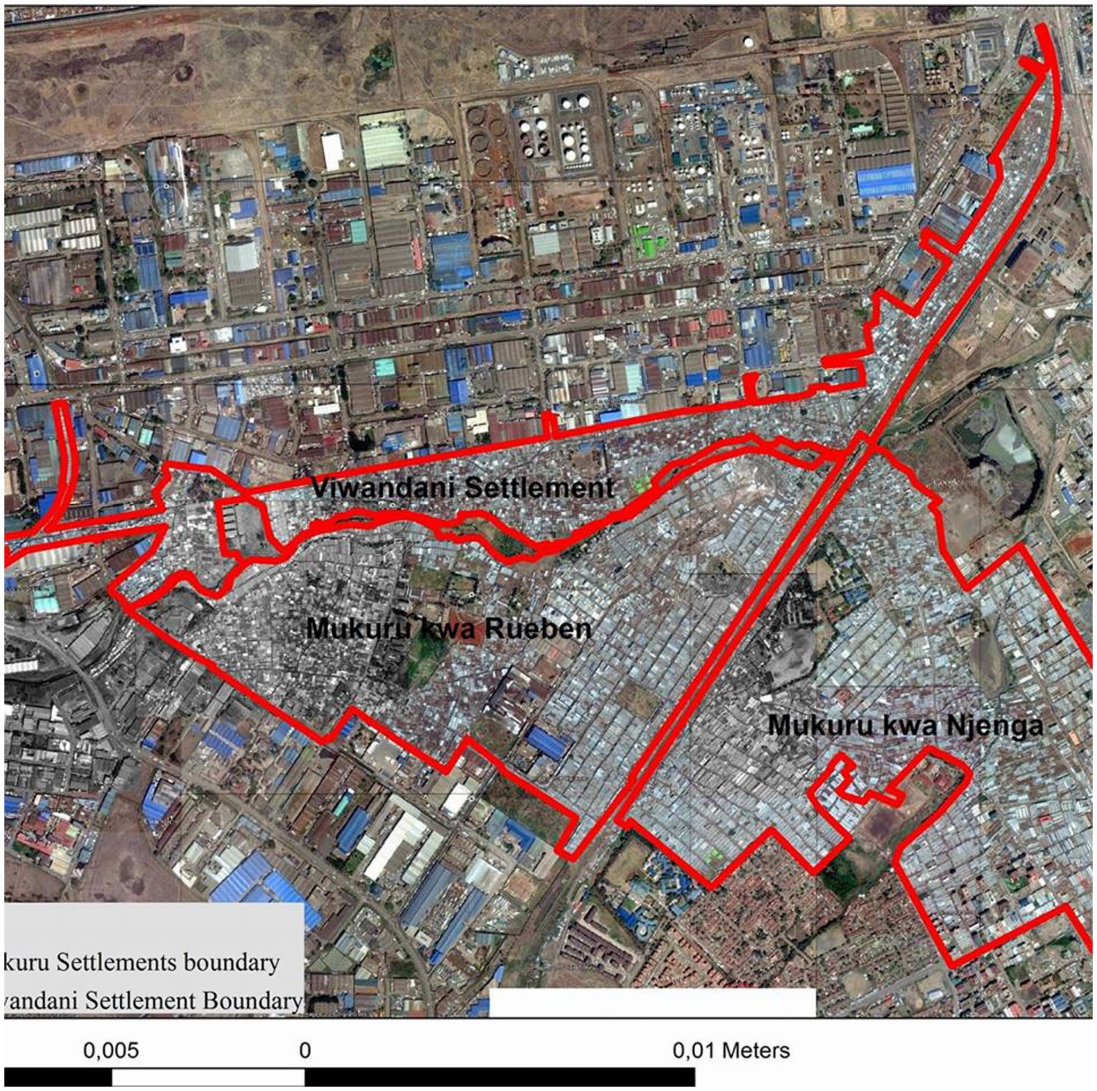
Study sites.

Poverty levels are at over 50.3% for the male and 49.7% for the women (20); It has an oil pipeline, high voltage electricity transmission lines and the highly polluted Nairobi River running through it. Mukuru was declared a Special Planning Area in 2017, leading to the development of the Mukuru Integrated Development Plan (20), as such the researchers explored CBS services; an alternative sanitation in informal settlements (Figure 1).

### 2.3 Target population

The population of interest were community members, local leaders, government officials for primary data, and researchers for reflections.

### 2.4 Sampling and sample size

Twenty-five (25) participants comprising 10 community members, 10 landlords/ structure owners (availability in the informal settlements), one local chief and four ministry officials working the sanitation sector were sampled for participation. Participants were selected by purposive sampling with a focus on maximizing diversity of background, experience, role, and period of stay. Community members selected ought to have resided in the study area for a longer period than the others in the same category to the best knowledge of the researchers (that was on averagely 15 years). Structure owners included in our study were those who had resided in the informal settlements for longer than the rest (on average 10 years). Saturation point was attained at the 10th community member and structure owner. Saturation is a point at which gathering more data about a construct reveals no new properties, nor yields any further insights (21). The study also included the only senior chief and four ministry officials in charge of sanitation. All participants included were willing to participate in the study.

### 2.5 Ethical considerations

A research permit from National Commission for Science, Technology and Innovation (NACOSTI), REF: NACOSTI/P/21/11328 was obtained. Before participating in an interview, all participants provided an informed written consent. The interviews were conducted in quiet spaces for privacy, confidentiality and for the quality of the audio files.

The ethical process was rigorously designed to address the highly sensitive nature of power-related data and mitigate the risk of participant retaliation, particularly when discussing actors like informal cartels. This was primarily achieved through layered consent and strict confidentiality protocols. We informed study participants in detail that their responses would be aggregated and anonymized, with identifying details (e.g., using pseudonyms for roles or locations) to ensure no single response could be traced back to them. For those discussing particularly sensitive topics, the option to use general statements rather than specific names were offered. Furthermore, interviews were conducted in private, secure, neutral locations outside the immediate settlement area whenever possible, reducing the risk of being observed by potential adversaries and establishing a safe space for participants to speak openly about the complex and power dynamics influencing sanitation services.

### 2.6 Data collection process

Data collection was done between September 2021 and February 2022 in Mukuru kwa Reuben informal settlements. Research assistants received training for 4 days on the aims of the study, data collection process, data collection tools, and research ethics. In-depth interview guides (IDIs) were used to collect data on CBS and the role of formal and informal actors in sanitation service delivery. Below is the description of data collection process:

Informal conversation was carried out between the participants and the researchers to find out key insights and to create rapport with the study participants before the IDIs. The concepts discussed enhanced probing during IDIs. Observation was done by the research assistants, which allowed for a holistic awareness of events as they unfold and as such, enabling more comprehensive understanding of what matters to respondents. These observations resulted in insights on what to probe in future IDIs.

In-depth discussions between the researcher assistants and the study participants were administered in pairs of 2 co-researchers; one who was moderating the interviews and the second, acting as an observer, note taker and facilitator of the recording of the conversations. The study reached saturation in the IDIs at the 25^th^ respondent and recruitment stopped. The outputs from informal discussions, observations, and reflective discussions informed and enhanced robust probing during IDIs.

### 2.7 Data management and analysis

Recorded audios from IDIs, reflections and informal discussions were translated and transcribed from Swahili to English and saved as individual Microsoft Word documents. Outputs (Table 1) were assigned number codes to prepare for analysis and to ensure confidentiality.

**Table 1:** Themes for analysis.

Themes	Sub-themes
Role of CBS in sanitation in informal settlements	Positive impact of CBS; cost and accessibility Public health safety of CBS Acceptability of CBS by community CBS supplements inadequate government initiatives CBS as a source of income
Formal and informal power structures related to CBS services	Formal power Informal power Powerless Both formal and informal power
Challenges facing CBS in informal settlements	*Governance and coordination issues* A lack of coordination among players Challenges on reporting due to inaccurate data *Financial and community constraints* Inadequate community involvement and consultations Low budgetary allocation to sanitation Sanitation cost
Strategies for enhancing sanitation in informal settlements	Government facilitation of CBS Embracing use of CBS by all Incorporating existing structures like manual pit empties

Transcripts were imported into NVivo 12 software for coding and analysis. NVivo is a qualitative data management software that can be shared and worked on in groups and facilitate thinking, linking, writing, modeling and graphing in ways that go beyond a simple dependence on coding (22). The final sample size of 10 landlords and 10 community members was validated by the qualitative principle of data saturation, which was reached when no new themes or insights emerged from subsequent interviews, confirming that the sample was sufficiently rich to address the study’s objectives, a practice that is widely accepted in qualitative research as the gold standard for justifying sample adequacy. The study used a framework analysis (23). The first step of framework analysis was listening to the recordings to familiarize the researchers with the information related to our objective. To ensure reliability, two researchers (an experienced qualitative researcher and an anthropologist) and four research assistants, who collected the data participated in the development of a coding framework by reading the outputs imported in NVivo 12 software, independently to establish an inter-coder agreement. An Inter-Coder Agreement test was performed on this subset, yielding an acceptable consensus score (e.g., a minimum of 80% agreement). All discrepant codes were systematically reconciled through consensus meetings and iterative discussions among the research team to refine the coding dictionary and ensure a shared, stable application of codes. This unified, validated codebook was then applied to the transcripts, providing a robust foundation for the final categorization and thematic development presented in the findings. Once the coding based on the agreed themes (Table 1) was complete, the two researchers proceeded with charting, mapping and interpretation of transcripts.

## 3 Results

### 3.1 Role of CBS in sanitation in informal settlements

Study participants described the CBS initially launched in 2010 to address the public health crisis of lacking sanitation in Mukuru’s informal settlements; they explained how Fresh Life Initiative by Sanergy provided a network of container-based toilets (Fresh Life Toilets) managed by local micro-entrepreneurs. These entrepreneurs maintained the toilets, collected user fees, and contributed to a sanitation solution that converted waste into fertilizer and energy. The initiative offered various service models for schools and residences, serving over 120,000 residents in Nairobi’s informal settlements with a focus on Mukuru, while also expanding services in other informal settlements.

*‘Fresh life has employed many youths here. They protect the CBS toilets (National Government-Local leader (Chief), Sept 15^th^ 2021).*

*‘Most people benefiting from simplified sewer projects are those living close to them or those living around accessible roads… freshlife is also helpful to residents far from the main roads.’ (Land Lord 3, Sept 16^th^ 2021).*

Structure owners alluded that CBS delivery model ensured safe fecal sludge management (FSM) along the entire service chain assuring public health safety. However, they suggested a need to consider the needs of the vulnerable in the community, including persons with disability (PWD). The participants also emphasized that when implemented and maintained, CBS systems can provide safe and sustainable sanitation solutions for communities, helping to improve public health and environmental conditions. As one landlord explained, [they] *collect the waste every day. We do not have to live with overflowing pits within our homes’, but there is need to consider the needs of the vulnerable population.*

*‘Fresh life staff are always with us here. If you experience a challenge relating to toilets, they will help you out.’ (Land Lord 6, Sept 17^th^ 2021).*

Participants’ positive reception of CBS was a testament to its effectiveness and appropriateness in addressing their sanitation needs. Structure owners highlighted that CBS had been well-accepted because providers performed their duties, such as emptying the containers, effectively and efficiently. They also noted that reliability in emptying and attention to proper maintenance by CBS providers contributed significantly to the community’s trust and acceptance of the system.

*“Fresh life is good to us. They also ensure emptying is done right. For example, no flies, no spillage, no delays” (Land Lord 4, Sep 16^th^ 2021).*

Local leaders also acknowledged how in the absence of government sanitation initiatives, other players stepped in to fill the service provision gap. And among them are service providers offering CBS. The study participants described how CBS systems can be quickly deployed in areas where government initiatives are slow to reach or where infrastructure is inadequate. Participants further illustrated how CBS could be implemented without extensive construction or plumbing infrastructure, making them ideal for areas with limited resources.

“*Fresh life provides sanitation facilities at the community in small spaces where sometimes it has been impossible for government to offer the service due to poor infrastructure, limited space, costs and lack of land ownership which make ordinary toilet adoption a challenge. They also ensure emptying is done right”* (*National Government-Local leader* (Chief), Sep 17^th^ 2021).

### 3.2 Power structures related to CBS services

Our findings revealed that cartels had taken control of access to sanitation services in informal settlements. These cartels dictated the cost, accessibility, and quality of these services, often providing substandard services at a high price. Despite widespread opposition to cartel operations, they had managed to fill the void left by inadequate government service delivery. Study participants noted that they often received poor-quality sanitation services that were overpriced, yet they relied on the services because of the lack of better alternatives. Operating as a group, cartels were reported to mediate sanitation services, driving up costs and limiting accessibility, as one participant noted. This intermediary role of cartels was illustrated to further entrench their power and impacted the residents’ daily lives.

*‘Cartels took over service delivery due to governments’ laxity, as people needed the services… ‘Cartels have zoned themselves and each cartel operate in its own area… They operate as a group because it consists of a powerful number of people’. (Land lord 4, Sep 16^th^ 2021)*

Informal power of community-based organization (CBO) initiatives was highlighted in how these groups operated and maintained sanitation facilities. Women and youth groups were managing public toilets, charging a small fee (3–5 shillings per use) to sustain their operations. These groups often collaborated with manual pit emptiers to ensure the cleanliness of the facilities. It was illustrated that these CBOs often lacked the financial capacity to support sanitation services at a larger scale. Their limited resources hindered their ability to undertake broader initiatives, such as awareness creation and extensive maintenance efforts. This highlights the need for additional support to empower these groups to sustain and expand their valuable sanitation efforts.

*‘Women and youth groups are currently running the public toilets. They charge people to use the facilities (3-5/ use). Some of these are clean because the groups organize with manual pit emptiers who provide the services (National Government-Local leader (Chief), Sept 15^th^ 2021)*

Government utilities underprovided the sanitation services, partly because they do not have a clear obligation to serve informal settlements and because of the fluid and rapidly urbanizing cities. Government and land lords acknowledged that it was not financially feasible for government utilities to extend mains or distribution lines to settlement communities and households under current. There were also limited government standards pertaining sanitation services available in the community.

*‘There are few or no government services {i.e., sanitation services}, due to financial constrains’ (Local government). ‘We don’t have any clear standards in place for sanitation management and faecal sludge derived products. We also need standards to regulate containment, emptying & transportation and treatment in informal settlements.’ (Policy actor-National government, Jan 18^th^ 2022).*

*Government interventions here have failed because of cartels. For instance; the government provided free electricity in the slums, cartels vandalized the meters and cartels were back with illegal connections. They have to remain in business. The risks of leaving naked electric wires laying on the ground, and passing through sewage drainages leads to deaths/fires… you may be electrocuted.’ (Land lord 1, Sept 16^th^ 2021).*

Results showed that underlying dynamics within both formal (government) and informal (community) structures in informal settlements significantly impacted access to sanitation. Cultural beliefs and taboos surrounding sanitation, as described by structure owners, community members, local leaders, and even government officials, made it a low priority and an embarrassing topic. These deeply ingrained cultural norms and religious perceptions hindered effective sanitation initiatives. Residents prioritized water and other basic needs due to both cultural beliefs and practical demands, leading to government funding often neglecting sanitation. This hidden power limits the opportunity for residents to access sanitation services. Study participants also described how leaders were also influenced by these biases, impacting sanitation policies.

‘*We face a complex challenge in informal settlements. Cultural sensitivities and ingrained beliefs surrounding sanitation make it difficult to prioritize and implement effective solutions. While we strive to address basic needs like water access, sanitation often falls behind due to limited resources and a lack of public demand’ (Policy actor-National government, Jan 18^th^ 2022).*

*“Because of the cultural practices of the communities, some do not accept using any products associated with toilets” (Land Lord 2, Sep 16^th^ 2022)*

*“Even us leaders have cultural issues, which define/dictate the direction we lead the Country in terms of sanitation provision…‘Women make better leaders in sanitation delivery than men, they understand the needs of all household members and assume maintenance/cleanliness roles. They have traditionally been mentored to be sanitation custodians.’ (Policy actor-National government, Jan 18^th^ 2022).*

Participants described how Landlords prioritized profits and deemed provision of toilets which required user fee in rental houses, as beneficial. As such, tenants had no option on sanitation services.

*‘It is difficult to know the owners of these plots. Most of the landlords have leased structures from cartels, and have to remit some money every month to them. To remain in business, they don’t provide toilets and when they do, they are connected to storm water drains. Constructing a unit rental structure that attracts monthly income is more beneficial than a toilet which will require emptying.’ (Land lord 1, Sept 16^th^ 2021).*

Study participants described how unclear ownership and responsibility were among major challenges to sanitation improvements within the community. Landlords and residents described a situation where no single entity took charge of sanitation services. This lack of clear ownership often resulted in cost-cutting measures by landlords, prioritizing profits over proper sanitation. As one community member expressed,

"*Landlords are unwilling to pay for manual pit emptying, looking to maximize their rent income… This approach led to risky practices of releasing faecal matter into open drains, jeopardizing public health’ (National Government-Local leader (Chief) Sept 15^th^ 2021)*

Further, land lords described how sanitation options were mainly through shared or private dry pit latrines which were unclean and uncovered, hence open defecation.

*‘The common sanitation option in this slum is pit latrine, which is mostly dirty and uncovered. It however presents another form of open defecation as the toilets are avoided.’ (Land lord 1, Sep 16^th^ 2021)*

For settlement residents, securing formal sanitation services was challenging due to household competing priorities for example on rent payment, food and clothes. Others include bureaucracies of acquiring title deeds, and sometimes cultural norms which disassociate people from their responsibility of ensuring safe excreta management. Unmet land tenure in the informal settlements affected sanitation innovations in informal settlements.

*‘Lack of land ownership limits expansion of structures and services. A minimum is provided to ensure survival. These people are needy, thus easily manipulated; politicians use them. Criminals hide here. They are armed, you can’t go for them. No serious project can be conducted here. You heard of destruction of public toilets during post-election violence in 2017? This is nobody’s land, recent road expansion projects demolished very many fresh life toilets.’ (National Government-Local leader (Chief) Sept 15^th^ 2021)*

*‘My space is leased. Cheap structures cushion me in case of evacuation.’ (Land Lord 10, Sept 17^th^ 2021).*

### 3.3 Challenges facing CBS in informal settlements

The analysis of the sanitation sector highlighted significant challenges stemming from governance and coordination issues, as well as financial and community constraints.

#### 3.3.1 Governance and coordination challenges

Challenges in the realm of governance was a distinct lack of coordination among players. Land owners/land lords alluded that lack of coordination among different players and consultation during implementation was a major factor, which limited the success of the slum upgrading initiatives, including sanitation initiatives. Participants from National government described challenges related to off-grid, data and reporting, community involvement and budgetary allocations. To start with, a lack of understanding on off-grid was among challenges in implementation initiatives, as the government had to provide a way forward. This fragmentation often hindered the effective implementation of sanitation projects and services.

*‘Recent road expansion projects by Nairobi Metropolitan Service have demolished many fresh life toilets… I think there was no consultation on the whole process’ (Land lord 7, Sept 17^th^ 2021).*

*‘There is lack of understanding {data} in the economics of the off-grid sanitation by the ministry, challenging implementation initiatives.’ (National government--Nairobi Metropolitan services).’ (National Government Policy actor Director (Water and sanitation services) Jan 18^th^ 2022).*

Furthermore, accurate oversight and strategic planning were compromised by challenges in reporting due to inaccurate data. There were reports of inaccurate data due to devolution of government functions to local levels and establishment of parastatals. Community members described reporting to be lacking or inadequate in transparency. This was described to possibly limit decisions, policies and interventions on sanitation, more so in informal settlements. The unreliability of reported data made it difficult to establish a clear baseline, monitor progress effectively, and ensure accountability across the sector

*‘Here I have not seen a proper way of reporting because some people {actor in sanitation space} just take matters upon themselves. They all know it all. Some are cons, they report wrong information’ (Community member 6, Sep 21^st^ 2021).*

#### 3.3.2 Financial and community constraints

Financial limitations posed a barrier, notably the low budgetary allocation to sanitation service delivery.

Beyond the financial issues, there was an observed deficit in grassroots engagement, characterized by inadequate community involvement and consultations. This lack of community-level participation and buy-in undermined the long-term adoption and maintenance of sanitation facilities, suggesting that solutions were often imposed rather than collaboratively developed.

National government acknowledged issues of low budgetary allocation and fragmented roles and responsibilities across different ministries, impacting sanitation efforts. Government officials illustrated how the ministry got inadequate budget for sanitation and water investment; thus, they prioritized water as a basic need. This indicated that limited funds forced ministries to focus on immediate necessities like water, often leaving sanitation underfunded. Furthermore, a lack of a clear definition of sanitation had led to funding being diverted primarily to water works and sewerage, and crucial aspects of sanitation were overlooked. Water utilities in Kenya were described to be curved around water provision and sewage treatment. This fragmentation hampered effective sanitation management and highlighted the need for a more integrated approach.

*‘Sanitation docket has progressively received low financing. The ministries do not get enough budget for sanitation and water investment; thus, they prioritize water as a basic need.’ (Policy actor-National government’). (National Government Policy actor Director (Water and sanitation services), Jan 18^th^ 2022).*

*‘The definition of sanitation has not been well covered thus the funding goes to the water works and the sewerage. We partnered in thinking that sanitation is sewerage. Water utilities are curved around water provision and sewage treatment.’ (Policy actor-National government PS Water and Irrigation, Feb 19^th^ 2022).*

*The sanitation docket is like an orphaned child; Roles and responsibilities are fragmented between various Ministries like Water and Sanitation (focuses more on water provision) and Health (hygiene). (National Government Policy actor Director (Water and sanitation services), Jan 18^th^ 2022).*

We also found that sustainability and scope of sanitation initiatives were constrained by underfunding, resulting in a persistent financial deficit where operational and maintenance costs exceeded available resources. This contributed to gaps regarding off-grid sanitation in informal settlements, which effectively shifted the responsibility and associated financial burden from public service to the individual household level. Consequently, access depended heavily on a user-fee model, creating an economic barrier. This burden was particularly acute for those relying on “pay-as-you-go” systems, which were found to be technically more expensive than other models. This structure indicated a regressive financial dynamic where residents with the least secure access faced the highest relative costs, underscoring a fundamental inequity in the urban sanitation economy.

*‘Residents have to pay rent before I organize for emptying. If not, it is difficult to get my money from the’ (Land lord 9, Sept 17^th^ 2021).*

*‘Fresh life toilets are expensive (850KES/Month). The Government should help us.’ (Landlord, 3)… ‘Some people here are spending 1000-2000 KES per month for their sanitation needs. This depends on the family size. Most people are charging 3-5KES per use’ (Land lord 1, Sept 16^th^ 2021).*

Community engagement in sanitation service delivery is key for ensuring project sustainability and accountability. There were consistent reports on low or no involvement of communities, yet, involving communities in generating the demand for sanitation facilities, their design and ongoing delivery can generally ensure that sanitation interventions are appropriate for the context and for users themselves.

*‘Sanitation decisions are made in government offices without involvement of the community. For example, they didn’t consider our views when they started other sanitation projects like simplified sewer system (Community Member 2, September 20^th^ 2021).*

*‘There is already a simplified sewer network project going on. This is already bringing problems of system blockage. People were not involved. They are required to pay 5,000 (Kenya shillings) connection fee. We are not prepared for this. Who will provide the toilet infrastructure? 5,000 is a lot of money for connection. The lines will be clogged with solid waste. Cartels have come up with plans to hack and connect illegally to that trunk.’ (National Government-Local leader (Chief), Sept 15^th^ 2021)*

### 3.4 Strategies for enhancing sanitation in informal settlements

Government officials who participated acknowledged that CBS delivery model can improve sanitation status within the slum. They expressed need for a sanitation team to facilitate container-based sanitation (CBS) services. The sanitation team was thought to spearhead CBS planning, and provide a space to connect members and partners as well as anyone interested in sanitation space, for improved sanitation services in informal settlements.

*“There also should be a sanitation team that ensures that container latrines are already in place to uphold sanitation as a part of the contingency plans’ (Policy Actor-National government PS, Water and Irrigation, Jan 18^th^ 2022).*

Government officials described how the use of CBS could be up-scaled by using the latrines in different places and all social classes; not only for the people who are regarded as “low class/poor/those living in slums” since this causes the resistance. Institutions can embrace the use of container latrines than flushable toilets which end up wasting a lot of water which could have been preserved if they embraced the use of container latrines. In terms of food security: waste can be managed to provide fertilizers and curb food insecurity. Government actors alluded that structures and institutions are in place that allow people at the grassroot to identify priority needs and eventual communication to the relevant authorities for action.

*Sanitation can be upscaled through advocacy for people to embrace the new sanitation technologies. (National Government Policy actor Director (Water and sanitation services), Jan 18^th^ 2022).*

*‘In the grass root levels, if a common man raises his concern {i.e., related to sanitation} to any relevant office, from the office he is guided to the institutions related to his need through the county and national levels.’ (National Government Policy actor Director (Water and sanitation services), Jan 18^th^ 2022).*

Some of the manual emptiers were registered and allowed to operate under occupational safety and health act guidelines. This was thought to facilitate the CBS service delivery as the emptiers would appreciate wearing of masks, gloves and protective clothing for their safety.

*‘We recruited and trained, and thereafter facilitated registration of manual pit emptiers who work here. We provided a transfer station under our mtaa fresh initiative’. (Freshlife staff-technical supervisor, Feb 9^th^ 2022).*

*“Communities need to learn to move away from their cultural behavior in order to embrace proper sanitation” (National Government-Local leader (Chief), Sept 15^th^ 2021).*

## 4 Discussion of findings

Our study illustrated the role of CBS in sanitation; formal and informal power structures; and challenges facing CBS and strategies for enhancing CBS. CBS was identified as a cost-effective and accessible solution to sanitation challenges in informal settlements, outperforming other initiatives like simplified sewer systems. CBS initiatives have positively impacted local economies by employing youth and providing sanitation services to residents far from main roads, who are often neglected by simplified sewer projects. The model ensures safe fecal sludge management, benefiting public health and catering to vulnerable groups, including persons with disabilities (PWD).

Community acceptance of CBS was due to its appropriateness, convenience, and the efficiency of service providers, who ensured daily waste collection and immediate assistance when challenges arose. Other studies have described the key role of CBS, illustrating how explicit recognition of CBS as improved sanitation in Kenya provides. It provides a platform upon which support for CBS approaches can be built, thus providing a strong basis for its development (16, 24). Appropriate regulations and strengthened enforcement mechanisms will be required to provide a level playing field on which CBS can compete with alternative approaches (4). Recent research also indicates that the true cost of CBS service delivery is lower than that of centralized sewerage (12, 19). The portable nature of CBS as a sanitation approach makes it appealing in these contexts as it required little space and limited or no in-house construction. In some cases, potential customers who lived in single-room dwellings have insufficient space to install a CBS household toilet, thereby rendering single-household CBS responses unfeasible. In such conditions, shared CBS approaches have emerged in Nairobi (4).

CBS was described to complement inadequate government initiatives by rapidly deploying services without the need for extensive infrastructure. Challenges reported in informal settlements included the role of cartels who controlled access and costs of sanitation services due to the government’s inadequate provision, a study done in Kenya in the water sector described how water provided by cartels is sometimes sold at prices 10 times higher than the official prices to which the formal providers must adhere (25), and could be the case for other services like most sanitation services in informal settlements. Community-based organizations (CBOs) also played a role, though they often lacked financial capacity to scale their initiatives, similar to other studies (10, 26). Formal government power was illustrated to have been constrained by financial and institutional limitations, with fragmented responsibilities and low budget allocations for sanitation. Hidden cultural biases and prioritization of other basic needs over sanitation further inhibited effective intervention. Inadequate coordination among stakeholders, inaccurate data reporting, limited community involvement, and high costs of sanitation services presented significant barriers. As such the main target market for CBS services was the urban poor, who typically lived in densely packed settlements, in rented accommodations, or with no formal land title hence facing compounded challenges in sanitation services (15, 26). There were instances of reported powerless status of community in the choice of sanitation options as a result of their inability to afford services of their choice, existence of cartels and other service providers who could make choices without consulting the community. This is depicted in other studies describing the powerless nature of residents in decision making affected by factors like ownership and financial capabilities (27, 28).

The study shows that cartels seized control of sanitation service delivery due to the residents’ urgent need for services. This is more than just criminal opportunism; it reflects a systemic failure of state capacity and legitimacy in informal settlements. By zoning themselves and operating as a powerful group, these cartels dictate high costs and substandard services, effectively mediating access and further entrenching residents’ powerlessness. This finding resonates strongly with studies across African cities like Nairobi, where the state’s failure to provide essential services like water and sanitation facilitates the rise of exploitative cartels that fill the gap, as noted by (36, 37). The inability of formal government interventions to succeed against this organized informal power evidenced by the cartels’ vandalism of free government electricity meters to maintain their illegal connections underscores that scaling CBS requires political will to disrupt established rent-seeking dynamics, not just technological innovation.

Results highlights a significant division of labor and responsibility in sanitation, noting that women and youth groups were running the public toilets and charging a fee for maintenance. Despite this vital informal role, these Community-Based Organizations are financially limited, hindering their ability to scale services, conduct awareness campaigns, or perform extensive maintenance (16, 19). This demonstrates and depicts a form of unrecognized governance: women and youth are relegated to labor-intensive, under-resourced sphere of operational management. This aligns with broader urban studies on unrecognized governance where women’s community efforts provide essential services but are rarely integrated or supported by formal municipal planning (29), thereby perpetuating a reliance on fragile, informal structures rather than building equitable, city-wide systems.

Strategies to enhance sanitation included government facilitation of CBS, embracing CBS across different social classes, incorporating manual emptiers, and advocating for cultural shifts toward improved sanitation practices. The strategies can be enhanced through training. However, training alone is not enough to build the capacity on CBS, as such what is also required is more information on the available financial systems and lower-cost appropriate technologies (26, 30). Addressing CBS related issues will require political will and resource allocation on the part of the Kenyan state and local country governments (24). Those working with and promoting CBS as a solution to technical sanitation challenges need to recognize the political and economic realities of the context and to work on building coalitions of action that begin to debate and clarify lines of responsibility and accountability for the provision of sanitation in informal settlements (24, 26).

## 5 Conclusion

This study provides valuable insights into the implementation of CBS in informal settlements, directly addressing the sanitation challenges faced in these areas. The findings highlight the potential of CBS to improve sanitation conditions, particularly in areas with limited infrastructure and resources. However, the success of CBS is contingent on addressing several challenges, including complex power dynamics, inadequate infrastructure, limited financial resources, and regulatory barriers. To maximize the impact of CBS, it is essential to strengthen partnerships between government agencies, private sector actors, and community organizations, invest in capacity building and training, and develop supportive policies and regulations. By addressing these challenges and leveraging the potential of CBS, it is possible to create healthier and more sustainable informal settlements.

In places where there are no regulations, rules should be created. For example, where a regulatory framework on CBS does not exist regarding issues like cost, safety and acceptability measures, interested actors in the sector should be develop a guiding framework. Establishing overall service standards could facilitate the broader replication of CBS service models and enable benchmarking of service quality, thus promoting consistency and further confidence in the CBS approach. Additionally, this would create a level playing field, allowing CBS approaches to develop alongside other sanitation services, particularly on-site sanitation (OSS) and FSM. Ultimately, the formalization by government entities and adoption of CBS benefit the consumer, offering better and more affordable service options.

Considering that users of CBS services are often resource-constrained households, among strategies for implementing the access to CBS should target subsidies and public-private partnerships to mitigate sanitation inequities. Funding structures based on sanitation coverage outcomes can increase accessibility of CBS services and strengthen urban sanitation systems. Further, there is a need for actors in the sector to formalize CBS within Nairobi’s sanitation master plan, creating targeted subsidies for low-income households, and regulating against exploitative cartels. The research concludes that scaling CBS successfully requires transcending purely technical considerations. The findings will contribute to policy-making in Nairobi’s Special Planning Area framework and broader debates on urban sanitation financing. Future implementation strategies should focus on establishing collaborative governance models and integrating local leadership to navigate and mitigate existing power dynamics, thereby addressing the systemic, contextual barriers required for achieving sustainable and equitable sanitation coverage.

To ensure the long term success of CBS, future studies should focus on several key areas. Studies should also investigate the most effective financial models, such as subsidies and public-private partnerships, to make CBS services more accessible and affordable for low-income households. There is a need to explore how to effectively build capacity on CBS and implement technologies with lower costs. Finally, research should aim to clarify the lines of responsibility and accountability for sanitation provision in informal settlements to better inform policy and resource allocation.

## Data Availability

The raw data supporting the conclusions of this article will be made available by the authors, without undue reservation.
